# Knowledge, Attitude, and Practice of Mothers regarding Oral Hygiene of Primary School children in Chennai, Tamil Nadu, India

**DOI:** 10.5005/jp-journals-10005-1535

**Published:** 2018-08-01

**Authors:** Deepa Gurunathan, Joyson Moses, Shanmugaavel K Arunachalam

**Affiliations:** 1 Professor, Department of Pedodontics, Saveetha Dental College Saveetha Institute of Medical and Technical Science, Saveetha University, Chennai, Tamil Nadu, India; 2 Professor, Department of Pedodontics, Thai Moogambigai Dental College Chennai, Tamil Nadu, India; 3 Senior Lecturer, Department of Pedodontics, Saveetha Dental College Saveetha Institute of Medical and Technical Science, Saveetha University, Chennai, Tamil Nadu, India

**Keywords:** Attitude, Knowledge, Mothers, Practice, Primary schoolchildren.

## Abstract

**Introduction:**

Children depend on their parents and caretakers for maintenance of their oral health. Parents play a major role in both preventive and treatment regime in these children. This study was conducted with the aim of evaluating the influence of parental education on knowledge, attitude, and practice of mothers regarding oral health of primary schoolchildren in Chennai.

**Materials and methods:**

A 15-item self-administered questionnaire was used among 465 parents of preschool children in Chennai, of which 432 mothers returned the filled questionnaire. The data collected were subjected to statistical analysis using frequency of responses and chi-square test (p < 0.01). Data were analyzed using statistical package.

**Results:**

Of the 432 mothers who participated in the study, 150 (35%) have studied up to school level and 282 (65%) have completed diploma/degree. The knowledge of mothers regarding the knowledge of importance of oral health for general health is appreciable, but the awareness of proper brushing habit, treatment of caries, and importance of dental visits is less in mothers who have school education when compared with graduate mothers.

**Conclusion:**

Mothers who are graduates are more aware of the importance of oral health in children, treatment of dental caries, and brushing technique than mothers with school education. Hence, it is essential that government and health care providers impart oral health knowledge to mothers, as they are the role-models for their children.

**How to cite this article:** Gurunathan D, Moses J, Arunachalam SK. Knowledge, Attitude, and Practice of Mothers regarding Oral Hygiene of Primary School children in Chennai, Tamil Nadu, India. Int J Clin Pediatr Dent 2018;11(4):338-343.

## INTRODUCTION

Parent’s belief toward children’s oral health plays an important consideration to improve children’s oral health. In spite of the fact that early childhood caries (ECC) has been widely studied and preventive programs implemented in certain countries,^1^ ECC is more commonly seen in socially disadvantaged groups, who are devoid of health care systems.^2^ Early childhood caries continues to be a social health problem in countries like in India where there is no national program of oral health assessment and primary oral health care.

In order to achieve the best oral health outcomes in children, parents are key persons in ensuring the well-being of young children.^3^ A number of risk factors are associated with ECC, which can be broadly classified into biological and social risk factors.^4^ Social risk factors comprise low parental education, low socioeconomic status, and lack of awareness about the dental disease.^5^ To maintain a good oral hygiene, both the parent and the child must work together. It is seen that poor attitude of parents generally reflect as a poor oral health in children and *vice versa^* The need to commence prevention at a very young age and that the best chance of reducing further inequalities in health relate to parents and in particular to mothers and children. Parents’ perceptions of their children’s oral health as being better than their own should not be mistaken for the children’s oral health status as being good.^2^ Hence, it is essential to assess the knowledge, attitude. and practices about their children’s oral health which will help the health providers to understand the reasons for development of oral diseases in children and failure to get them treated.

## MATERIALS AND METHODS

The subjects of this study were selected randomly from a public school, SBOA Matriculation School. A total of 465 students were selected for this study and each of them was provided with a questionnaire for the study, and each of the questionnaire contained 15 questions. Subjects were parents of children from classes 1 to 5.

Approval from the directorate of education was obtained, and a letter was sent to the selected school explaining the purpose of this study and the procedures that would be followed during its conduct. The principal of the school was asked to inform the students and their parents about the study, and a day was set for distributing and collecting the data.

The questionnaire was divided into three categories. The dental attitude, knowledge, and practice of mothers regarding their children’s oral health were assigned in each division.

## RESULTS

A total of 465 students belonging from classes 1 to 5 were selected randomly and their mothers were asked to answer the questionnaire. Out of 465 students, 432 returned completed questionnaires with a response rate of 97.7%. Out of these, mothers who have completed school were 34.72% and mothers who have completed diploma/ degree were 65.28%. Of the students, 57% were males and 43% were females. Of the total population, mothers who have done only schooling were 150 (35%) and who have completed diploma/degree were 282 (65%).

### Knowledge of Mothers regarding Oral Health of Children

Various questions regarding the oral health knowledge, such as how important healthy teeth are for general health, reasons for brushing teeth, how often should children have dental check-up were asked; 96.0% of mothers who completed school accepted that healthy teeth are important for general health and 4% did not even know how important healthy teeth are for general health. Among the mothers who completed diploma/degree, 96.8% agreed and 2.1% were unaware that healthy teeth are important for general health (Table 1).

In all, 43.9% of mothers who completed school stated cleaning as a reason for brushing teeth, 42.1% for caries prevention, and 14.0% stated that brushing is done to prevent foul breath. Cleaning (48.9%) was the major reason stated by the mothers who completed diploma/ degree; 44.7% suggested that brushing is done for caries prevention and 6.4% said that brushing was done to prevent foul breath.

**Table 1:** **Table 1:** Knowledge of mothers regarding oral health of children

				*Graduation*					
*Questions*				*School*		*Diploma/graduate*		p-value*	
Healthy teeth are very important for general health		Agree		48		91		0.623	
		Disagree		0		1			
		Don’t know		2		2			
It is possible to prevent the teeth loss due to caries		Agree		32		71		0.328	
		Disagree		7		10			
		Don’t know		11		13			
Children’s teeth should be checked regularly by dentist		Agree		40		77		0.534	
		Disagree		5		12			
		Don’t know		5		5			
If yes once in every		3 months		30		26		0.009*	
		6 months		17		42			
		12 months		10		26			
Reasons for brushing teeth		Bright teeth		25		46		0.288	
		Prevent decay		24		42			
		Foul breath		8		6			
What are the main sources of dental health information?		Dentist		32		74		0.037	
		Physician		1		5			
		Media		7		15			
Bacteria and sugar can cause tooth decay		Agree		42		83		0.769	
		Disagree		2		2			
		Don’t know		4		9			
Bacteria and dental plaque cause gingival bleeding		Agree		34		64		0.909	
		Disagree		3		4			
		Don’t know		14		26			
Preventive measures of dental diseases		Toothbrushing		35		70		0.064	
		Fluoride toothpaste		8		21			
		Floss		3		3			
		Pit and fissure sealants		2		0			
		Fluoride application		2		0			

A total of 80% of those who completed school agreed that children should have a regular dental check-up, 10% disagreed with the same, and 10% did not even have knowledge about it. And among the mothers who have completed diploma/degree, 81.9% agreed to it; 12.8 and 5.3% disagreed.

### Attitude of Parents regarding Oral Hygiene of Children

The questionnaire was prepared to know the attitude of parent toward oral hygiene, and five question were given; 42% of mothers who have done schooling stated that they do not care if no pain in a decay tooth, 32% said they would just try to cope with it, 20% would see the dentist. While 85% of mothers who completed diploma/degree said that they would see the dentist (Table 2).

A significant difference was seen with respect to the attitude of the parents whose education was diploma and schooling. Parents who were graduated or held diploma felt that it is necessary to teach the children brushing and take the children to dentist if they have signs of tooth decay. Also most of the graduate parents (46.8%) have reported rolling as the method of toothbrushing.

No significant difference was seen among the interval of exchange of toothbrush and the frequency of dental visits.

### Practice of Mothers regarding Oral Hygiene of Children

Among the day-to-day practices, parents with diploma or graduation (40%) checked regularly their child’s teeth after toothbrushing than the parents who have done only schooling. No significant difference was seen among the parents of diploma and school graduated among the frequency, occasion, and time spent on toothbrushing (Table 3).

## DISCUSSION

The study was done to evaluate the influence of parental education on knowledge, attitude, and practice of caretakers regarding the oral health of children. Parents are the role-models for their children, and the habits adopted during childhood when the child is totally dependent on the mother are powerful means of establishing a novel behavior in children, such as that of toothbrushing.^7,8^ Hence, parents themselves have to have a good knowledge and attitude toward oral health in order to instill good oral habits in their children.^9,10^ In addition, the good knowledge and attitude must translate into good oral hygiene and dietary practices in order to have beneficial effects on dental health.^11^

Parental knowledge about infant oral health was found to be lacking in many studies.^12,13^ It was seen in the present study that education of parents plays a vital role regarding the attitude, knowledge, and practice of oral health in their children. A significant difference was seen among the response of educated and uneducated parents in regard to brushing habit, reason for gingival bleeding, and measures taken in order to treat dental problems. Hence, mothers with higher education have good knowledge, attitude, and practice about the oral health of children, which is similar to studies done in Kuwait.^14^

**Table 2:** **Table 2:** Knowledge of mothers regarding oral health of children

				*Graduation*					
*Questions*				*School*		*Diploma/graduate*		p-value*	
It is necessary to teach children toothbrushing		Agree		32		85		<0.001*	
		Disagree		18		9			
Till what age, children need help from adults in toothbrushing?		5 years		26		36		0.274	
		10 years		17		43			
		15 years		7		15			
If having signs of tooth decay what do you do?		Don’t care		21		9		<0.001*	
		Try to cope up		16		0			
		Visit dentist		10		80			
		Brush teeth		3		5			
Frequency of visiting dentist		6 months		23		41		0.143	
		12 months		10		34			
Method of brushing		Horizontal		27		20		<0.001*	
		Vertical		11		30			
		Rolling		12		44			
Interval of exchange of toothbrush		Less than a year		40		69		0.347	
		More than a year		6		20			
		Don’t know		4		5			

*p < 0.05

**Table 3:** **Table 3:** Practice of mothers regarding oral health of children

						*Graduation*					
*Questions*				*Knowledge/attitude/practice*		*School*		*Diploma*		p-value*	
Frequency of toothbrushing		Once daily		Practice		20		32		0.479	
		Twice daily				30		62			
Occasion of toothbrushing		Morning		Practice		34		63		0.865	
		Evening after meals				8		13			
		Evening after sweets				8		18			
I check my child’s teeth after brushing		Regularly		Practice		25		40		0.026*	
		Sometimes				8		42			
		Never				7		12			
Time spent on brushing teeth		<3 minutes		Practice		29		50		0.581	
		>3 minutes				21		44			
Dental visits for the child within the past		Once		Practice		17		42		0.235	
12 months		Twice				7		18			
		Thrice				5		10			
		Never				21		24			

*p-value calculated using chi-square test; p < 0.05 is statistically significant

Though there was no statistically significant difference between the two groups regarding the knowledge of healthy teeth toward general health, the graduate mothers were more concerned about the oral health, which is similar to previous studies.^14,15^ It is difficult to employ effective disease preventive strategies without basic knowledge of caries risk factor and oral mainte-nance.^16^ Majority of the parents had knowledge that sweetened food substances cause caries. The findings of the present study were similar to findings of Suresh et al,^16^ Lin et al,^17^ and Kumar et al,^18^ who also reported that parents of preschoolchildren had good knowledge about dietary process.

In spite of a substantial number of participants being aware that preventive measures can prevent dental diseases, there are shortcomings in the area of preventive practices. The school literate mothers did not give importance to brushing of teeth which is the basic preventive tool. The results are in agreement with the study by al-Tamimi and Petersen^19^ who reported a diffuse oral health knowledge and positive attitude of mothers toward prevention and participation in oral health education of children. A study by Hood et al^11^ in the United Kingdom also showed similar results where parents were well informed about the causes of caries, but were less sure of preventive methods. In the present study, others are aware of preventive measures, such as fluoride, floss, and sealants, but the extent to which these tools are used is questionable.

Parisotto et al^20^ showed that the higher the levels of maternal salivary mutans streptococci (MS), the greater the risk of transmission of MS to their infant. Apart from MS salivary levels, the mother’s oral hygiene, snack frequency, periodontal disease, and socioeconomic status are also associated with infant colonization.^21^ Toothbrushing is an effective tool in maintaining the oral hygiene and prevention of caries. In the present study, importance of brushing was underestimated by parents who had only school education when compared with graduates which is also seen in studies done by Astrom^22^ and Vallejos-Sanchez et al.^23^ However, most of the mothers felt that brushing helps to have clean teeth and to prevent caries. Hence, the mothers do understand the advantage for brushing, but not able to appreciate the importance of daily brushing. Most of the mothers agreed that till 5 years, brushing of children needed to be supervised which is similar to a study done in Mangaluru.^24^ Mothers with higher education felt that it is necessary to teach children toothbrushing and check the teeth after children brushed their teeth. This could motivate the children to do better brushing and to maintain the oral hygiene.

In addition, mothers with lesser education are not aware of the reason for bleeding gums. Hence, parents with only school education might not understand the importance of brushing. This can lead to reduced frequency in brushing in children which is not reflected in the study. However, Martins et al^25^ have reported that there is a difference between reported toothbrush-ings. This is supported by the observation in our study that even if mothers say that the children brush twice daily, when probed the occasion of brushing, they have responded as brushing was done only in the morning. Most of the participants in the study knew that the child’s brushing should have been supervised till the age of 5 years; however, over half the parents in rural Victoria Gussy were of the opinion that children were capable of brushing their own teeth by the age of 4 years. In another study by Vaitkeviciene et al,^26^ a very low percentage of parents guided and brushed their children’s teeth.

Mothers with higher education in this study knew about the rolling and vertical method of brushing unlike school-educated mothers. This proves that mothers with higher education are accessible to information regarding oral hygiene which is in accordance with a study done in Norway.^22^

In the present study, there was no statistically significant difference between parents with higher education and those with school education. However, although many parents reported taking their children to the dentist every 6 months, most respondents only take their children to the dentist when a problem arises, which is similar to a study done in Brazil.^27^

It has been observed in this study that most of graduated mothers derive knowledge regarding oral health of their children from the dentists which is similar to findings in a study done in Brazil.^27^ Mothers who visit the dentist often may acquire more knowledge regarding the oral care of children.^28^

Thus, it can be concluded form this study that mothers who have school education are not much aware of the preventive measures of dental disease, importance of toothbrushing, and dental visits. Hence, it is the responsibility of the government and health care providers to impart oral health knowledge to mothers, as they are the role-models for their children.
